# Body composition parameters correlate with the endoscopic severity in Crohn’s disease patients treated with infliximab

**DOI:** 10.3389/fnut.2023.1251448

**Published:** 2023-08-21

**Authors:** Wuli Tang, Gang Xie, Junlin Li, Wei Tan, Rongqi Yi, Ling Yang, Lingqin Zhang, Jiaxing Deng, Yue Zhang, Kang Li

**Affiliations:** ^1^Chongqing Medical University, Chongqing, China; ^2^Chongqing Institute of Green and Intelligent Technology, Chinese Academy of Sciences, Chongqing, China; ^3^Chongqing School, University of Chinese Academy of Science, Chongqing, China; ^4^Department of Radiology, Chongqing General Hospital, Chongqing, China; ^5^Department of Radiology, The Third People’s Hospital of Chengdu, Chengdu, China; ^6^Department of Gastroenterology, Chongqing General Hospital, Chongqing, China; ^7^Department of General Medicine, Chongqing General Hospital, Chongqing, China

**Keywords:** Crohn’s disease, body composition, disease behavior, disease activity status, malnutrition

## Abstract

**Background:**

The disease activity status and behavior of Crohn’s disease (CD) can reflect the severity of the disease, and changes in body composition are common in CD patients.

**Aims:**

The aim of this study was to investigate the relationship between body composition parameters and disease severity in CD patients treated with infliximab (IFX).

**Methods:**

Patients with CD assessed with the simple endoscopic score (SES-CD) and were treated with IFX were retrospectively collected, and body composition parameters at the level of the 3rd lumbar vertebrae were calculated from computed tomography (CT) scans of the patients. The correlation of patients’ body composition parameters with disease activity status and disease behavior was analyzed, and the diagnostic value of the relevant parameters was assessed using receiver operating characteristic (ROC) curves.

**Results:**

A total of 106 patients were included in this study. There were significant differences in the subcutaneous adiposity index (SAI) (*p* = 0.010), the visceral adiposity index (VAI) (*p* < 0.001), the skeletal muscle mass index (SMI) (*p* < 0.001), and decreased skeletal muscle mass (*p* < 0.001) among patients with different activity status. After Spearman and multivariate regression analysis, SAI (*p* = 0.006 and *p* = 0.001), VAI (*p* < 0.001 and *p* < 0.001), and SMI (*p* < 0.001and *p* = 0.007) were identified as independent correlates of disease activity status (both disease activity and moderate-to-severe activity), with disease activity status independently positively correlated with SAI and SMI and independently negatively correlated with VAI. In determining the disease activity and moderate-to-severe activity status, SMI performed best relative to SAI and VAI, with areas under the ROC curve of 0.865 and 0.801, respectively. SAI (*p* = 0.015), SMI (*p* = 0.011) and decreased skeletal muscle mass (*p* = 0.027) were significantly different between different disease behavior groups (inflammatory disease behavior group, complex disease behavior group) but were not independent correlates (*p* > 0.05).

**Conclusion:**

Body composition parameters of CD patients treated with IFX correlate with the endoscopic disease severity, and SMI can be used as a reliable indicator of disease activity status.

## 1. Introduction

Crohn’s disease (CD) is an inflammatory bowel disease that can involve any part of the gastrointestinal tract and is characterized by alternating relapses and remissions, which can result in a variety of complications, such as intestinal strictures, perforations, and fistulas, severely reducing the quality of life of patients (1). During the course of chronic inflammatory disease in CD patients, 65–75% of patients may experience malnutrition due to dietary restrictions and impaired digestion and absorption (2), while the chronic malnutrition status may lead to an imbalance of the intestinal flora and further exacerbate the inflammatory response of the intestine (3). In recent years, attention to the nutritional status of patients to reduce the risk of malnutrition and improve the quality of life of patients has gradually received attention from IBD experts (4).

The malnutrition status of CD patients can be characterized by decreased muscle mass or altered fat distribution (5). The intestinal inflammatory response can cause translocation of enteric bacteria to the peri-mesenteric adipose tissue and significantly promote mesenteric adipose tissue hyperplasia (6). At the same time, inflammatory factors such as tumor necrosis factor-α (TNF-α) released from the intestinal inflammatory response and proliferating adipose tissue can activate the NF-kb signaling pathway to reduce muscle protein formation and promote muscle protein degradation by the expression of MURF-1 and atrogin-1, resulting in reduced muscle mass in CD patients (7). Thus, it is clear that the intestinal inflammatory response is a driver of body composition changes in CD patients. In turn, quantitative monitoring of body composition changes in CD patients may be useful to reflect the severity of inflammation (disease activity status and disease behavior).

The use of computed tomography (CT) image data for body composition assessment is feasible. Previous studies evaluating the impact of body composition parameters on clinical prognosis in patients with CD based on CT or magnetic resonance imaging (MRI) have shown that changes in body composition are associated with poor prognosis in patients (8–13). Additionally, different drug treatments may cause changes in body composition distribution in CD patients (14–17). For example, a previous study showed a link between infliximab (IFX)-induced treatment and increased total abdominal fat in patients. Shen et al. (17) suggested increased visceral fat in IFX-treated patients was associated with attenuated mucosal healing. And Lim et al. (16) indicated that IFX, but not ADA trough levels, were significantly correlated with body composition-related parameters. Since IFX is the most widely used drug in clinical treatment (14), so to clarify the relationship between body composition changes and disease severity in CD patients and to exclude the interference of different drug treatments on the study results, only patients treated with IFX were included in this study to further verify the clinical value of body composition changes. As the endoscopic score is the gold standard for assessing the activity of CD (18), clarifying the correlation between body composition changes and endoscopic severity in patients with CD may help guide clinical optimization of disease management and timely adjustment of medication dosage to delay disease recurrence and prevent disease complications, thus further improving patients’ quality of life.

## 2. Materials and methods

### 2.1. Research subjects

In this study, patients with CD who met the following inclusion and exclusion criteria were retrospectively analyzed from December 2019 to April 2023. Inclusion criteria: (1) Patients diagnosed with CD based on a comprehensive assessment that included endoscopic, imaging, pathological, and clinical presentation (19, 20); (2) All patients received more than 3 consecutive doses of IFX prior to CT scan and simple endoscopic score for CD (SES-CD); (3) Patients who underwent SES-CD within 3 days of CT scan; (4) patients with clear imaging and complete clinical data. Exclusion criteria: (1) patients with a recent history of abdominal surgery; (2) patients with combined malignancy and other metabolic diseases.

This study was approved by the Medical Ethics Committee of Chongqing General Hospital (registration number: KY S2022-092-01), and written informed consent was waived. All procedures were performed in compliance with the Declaration of Helsinki.

### 2.2. Clinical data collection

General demographic data of the subjects were collected, including age, sex, height, weight, body mass index (BMI), disease duration, and smoking status, while laboratory test data, including C-reactive protein (CRP), albumin, and erythrocyte sedimentation rate (ESR), were recorded within 3 days of the subjects’ imaging examinations. According to the Montreal classification (21), in this study, nonstenotic and nonpenetrating (B1) were defined as inflammatory disease behavior, and stenotic (B2) and penetrating (B3) were defined as complex disease behavior. In addition, adequate bowel preparation is crucial for successful endoscopy in patients with CD (22), and all patients in this study underwent adequate bowel preparation before endoscopic examination. An experienced gastroenterologist assessed the ulcer size, area, extent of lesion, luminal stenosis and degree by endoscopy, and scored each segment of the ileum and colon according to SES-CD. The sum of all parameters in each bowel segment was the total SES-CD score, ranging from 0 to 56 (23). Based on previous studies (24, 25), we defined the degree of disease activity in CD patients according to the SES-CD score as remission group (≤2 points), mild activity group (3–6 points), and moderate-to-severe activity group (≥7 points).

### 2.3. CT examination methods

A Philips IQon spectral CT scanner was used to scan and enhance the abdomen of CD patients with the following parameters: tube voltage of 120 kV, tube current of 115 mA, slice thickness and slice spacing of 1.0 mm, and field of view (FOV) of 410 mm × 410 mm. Quality control was performed monthly using a Quantitative CT (QCT) body film (Model 4, QCT pro, mindways, United States) and an image analysis system based on the original image of the CT scan body film (Model 4).

### 2.4. Body composition measurements

**Figure 1:**
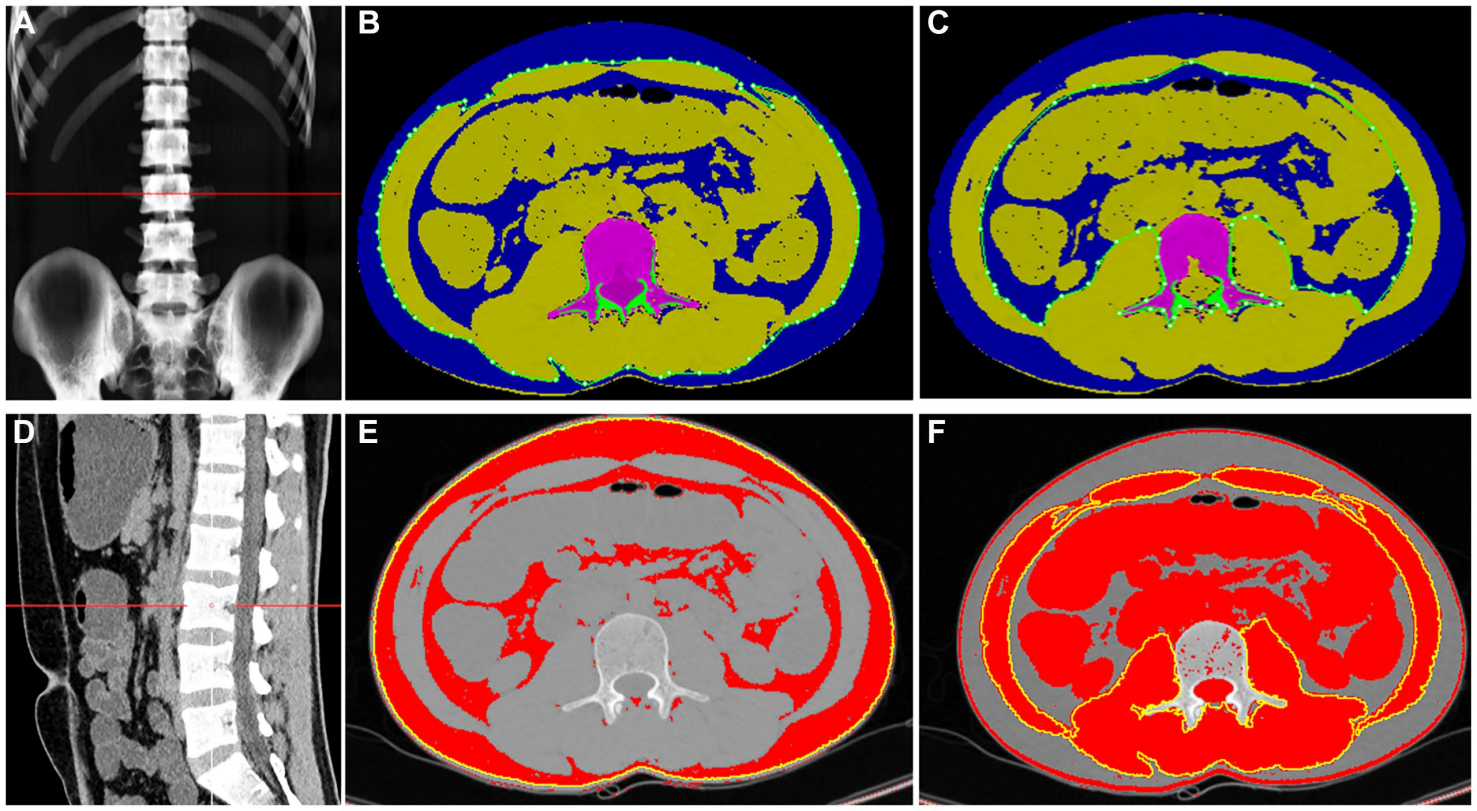
The QCT pro **(A–C)** and ImageJ **(D–F)** diagrams of body composition measurements of the same patient through the middle portion of the 3rd lumbar vertebra. The QCT pro automatically identifies and calculates the SFA and VFA **(B,C)**, which are shown in blue in the **(B,C)**; and the SMA is the total area in the green aperture of **(B)** minus the total area in the green aperture of **(C)**, indicated by the partially yellow area in **(B,C)**. ImageJ software semi-automatically measured SFA, VFA, and SMA at different body composition thresholds **(E,F)**. In **(E)**, the red color represents SFA and VFA, while in **(F)**, the red area within the yellow circle represents SMA.

We selected axial images at the mid-level of the 3rd lumbar (L3) vertebral body on the CT scan for body composition measurements. This is because previous studies have shown the strongest correlation between body composition parameters at this level with whole-body components (26, 27). The body composition of the patients was measured by the same investigators using QCT pro and ImageJ software (Figure 1). The CT scans were directly transferred to the QCT pro workstation for automated body composition measurements. Meanwhile, single slice tomographic images at the level of L3 were imported into ImageJ software to semiautomatically outline visceral fat area (VFA), subcutaneous fat area (SFA), and calculate total fat area (TFA), which is the sum of SFA and VFA, according to the fat threshold - 150 to −50 HU; skeletal muscle area (SMA) was outlined according to the skeletal muscle threshold −30 to +150 HU. The above body composition parameters were normalized for height squared (m^2^) to obtain the total adiposity index (TAI), subcutaneous adiposity index (SAI), visceral adiposity index (VAI) and skeletal muscle mass index (SMI). The criteria for defining the decreased skeletal muscle mass in this study were SMI < 52.4 cm^2^/m^2^ in men and SMI < 38.5 cm^2^/m^2^ in women (28), and visceral obesity was defined as VFA > 130 cm^2^ (29).

### 2.5. Statistical analysis

Statistical analyses were performed with SPSS Statistics 25.0. Normally distributed measurements are herein expressed as the mean ± standard deviation ($\bar{x}\pm s$), and comparisons between groups were made using the independent samples *t* test or one-way ANOVA. The bias distribution of the measurement data is expressed as the median (25, 75%), and the Mann–Whitney test or Kruskal–Wallis test was used for comparison between groups. Count data are expressed as cases (%) and were compared between groups using the χ^2^ test or Fisher’s exact probability method. The Bonferroni method was used for two-way comparisons of multiple data groups. The intraclass correlation coefficient (ICC) was used to assess the agreement between measurements of body composition parameters by the same physician using two methods, with ICC > 0.75 indicating good agreement of measurements. Body composition parameters measured by QCT pro were included in the statistical analysis. Demographics, laboratory data, and body composition parameters were subjected to Spearman correlation analysis, the correlation coefficient matrix was plotted, and a multivariate analysis was performed using binary logistic regression. Receiver operating characteristic (ROC) curves were used to evaluate the diagnostic efficacy. *p* < 0.05 was considered to indicate a statistically significant difference.

## 3. Results

### 3.1. Basic subject characteristics

**Figure 2:**
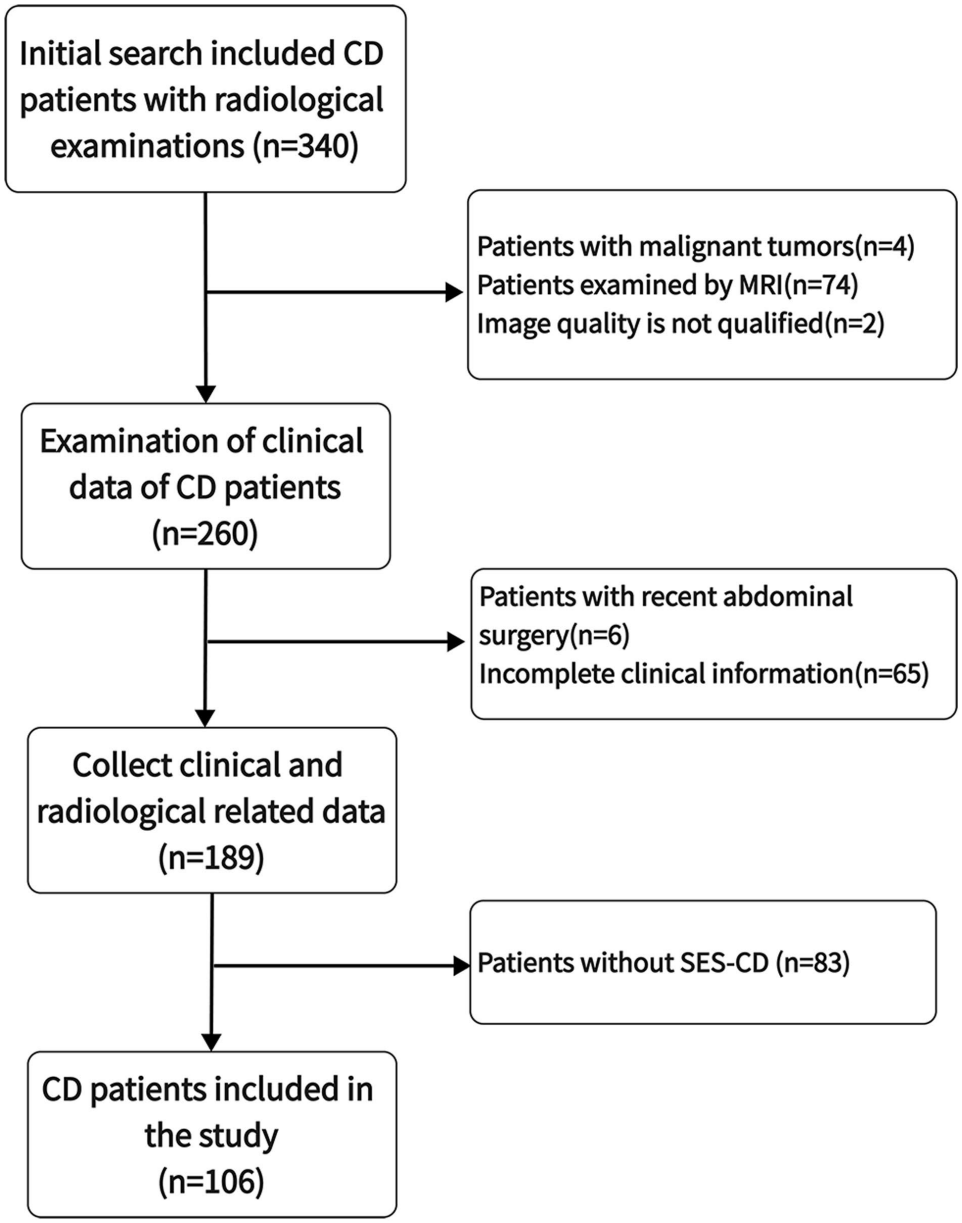
Selection process for research subjects.

A total of 340 CD patients with CT examinations between December 2019 and April 2023 were initially screened, and 106 CD patients were finally included after the application of strict inclusion and exclusion criteria for eligibility (Figure 2). Sixty-eight (64.15%) of the patients were male, the mean age was 26.18 years (range 9–59 years), the mean disease duration was 3.41 years (range 0.17–15 years), the mean BMI was 20.43 kg/m^2^ (range 13.01–33.06), 26.42% of patients were underweight (BMI < 18.5 kg/m^2^), and 11.32% of the patients were overweight (BMI > 24 kg/m^2^). The median SES-CD activity score was 5 (interquartile range 2–11), with 72 patients (67.92%) having active disease and 64 patients (60.38%) exhibiting complex disease behavior (Table 1).

**Table 1:** Baseline characteristics of the patients.

Variables	Total
*n*	106
Male	68 (64.15%)
Height (m)	1.66 ± 0.84
Weight (kg)	56.43 ± 11.29
BMI (kg/m^2^)	20.43 ± 3.42
BMI < 18.5 kg/m^2^	28 (26.42)
BMI > 24 kg/m^2^	12 (11.32)
Age at the time of CT examination (year)	26.18 ± 7.91
Disease duration (years)	3.41 ± 2.75
**Montreal classification**	
**Age (years)**	
≦16 (A1)	6 (5.66)
17–40 (A2)	96 (90.57)
>40 (A3)	4 (3.77)
**Location**	
Ileal (L1)	16 (15.09)
Ileocolonic (L2)	9 (8.49)
Colonic (L3)	81 (76.42)
**Behavior**	
Nonstricturing/penetrating (B1)	42 (39.62)
Stricturing (B2)	58 (54.72)
Penetrating (B3)	6 (5.66)
Perianal (P)	75 (70.75)
Serum Alb (g/l)	42.76 ± 5.12
CRP (mg/l)	4.49 (0.95, 11.51)
ESR (mm/h)	11.50 (6.00, 29.00)
SES-CD	5 (2.00, 11.00)
Smokers	4 (3.77)
Decreased skeletal muscle mass (M/F)	49 (46.23)/29 (27.36)
Visceral obesity (M/F)	12 (11.32)/8 (7.55)

Values are presented as N (%), the mean ± SD, or the median (interquartile range). BMI, body mass index; Alb, albumin; CRP, C-reactive protein; ESR, erythrocyte sedimentation rate; SES-CD, simple endoscopic score for Crohn’s disease; M, male; F, female.

### 3.2. Measurement of body composition-related parameters

The SFA, VFA, and SMA measured with QCT pro and ImageJ, respectively, showed a high degree of agreement with ICC values >0.95 (Supplementary Table S1). According to the measurements obtained from QCT pro, the average values of TAI, SAI, VAI, and SMI were 68.97 ± 20.56, 32.88 ± 14.41, 35.82 ± 11.43, and 39.42 ± 7.55, respectively. A total number of 78 patients (73.58%) had decreased skeletal muscle mass, of which 49 (46.23%) were male patients, and 20 patients (18.87%) had visceral obesity, of which 12 (11.32%) were male patients (Table 1).

### 3.3. Differential analysis of body composition parameters of CD patients In different disease activity status

According to the SES-CD score, there were 34 (32.08%), 28 (26.41%), and 44 (41.51%) patients in the remission, mild activity, and moderate-to-severe activity status, respectively, with no significant differences in demographic data among the different activity statuses (*p* > 0.05). Laboratory data were significantly different among the three statuses (ESR: *p* = 0.001; ESR、albumin: *p* < 0.001), and with increasing disease activity, CRP and ESR increased while albumin levels decreased (Table 2).

**Table 2:** Comparison of clinical data and body composition parameters in different activity status of CD patients.

Variables	Remission (*n* = 34)	Mild activity (*n* = 28)	Moderate-to-severe activity (*n* = 44)	*p* value
**Demographic data**				
Male Gender	21 (61.76)	16 (57.14)	31 (70.45)	0.486
Age (years)	27.24 ± 7.95	25.39 ± 8.83	25.86 ± 7.36	0.626
BMI (kg/m^2^)	20.64 ± 2.90	20.80 ± 4.27	20.03 ± 3.22	0.589
Disease duration (years)	3.83 ± 3.22	3.56 ± 3.24	3.00 ± 1.90	0.403
Location				0.059
L1	8 (23.53)	6 (21.43)	2 (4.55)	
L2	4 (11.76)	3 (10.71)	2 (4.55)	
L3	22 (64.71)	19 (67.86)	40 (90.90)	
Behavior				0.386
B1	17 (50.00)	12 (42.86)	13 (29.55)	
B2	15 (44.12)	14 (50.00)	29 (65.90)	
B3	2 (5.88)	2 (7.14)	2 (4.55)	
Perianal (P)	22 (64.71)	21 (75.00)	32 (72.73)	0.629
**Laboratory data**				
ESR (mm/h)	6.5 (4.00–16.50)	11.50 (6.00–22.75)	20.00 (7.25–38.50)	**0.001**
CRP (mg/l)	1.00 (0.34–3.53)	3.49 (0.88–7.58)	11.81 (4.67–37.04)	**<0.001**
Serum Alb (g/l)	45.44 ± 3.83	43.39 ± 4.14	40.29 ± 5.46	**<0.001**
**Body composition**				
SAI (cm^2^/m^2^)	38.41 ± 13.29	34.22 ± 17.52	28.20 ± 13.48	**0.010**
VAI (cm^2^/m^2^)	29.92 ± 10.83	34.07 ± 13.16	42.14 ± 9.67	**<0.001**
TAI (cm^2^/m^2^)	68.33 ± 19.86	68.29 ± 27.77	70.98 ± 21.57	0.839
SMI (cm^2^/m^2^)	46.19 ± 8.58	38.92 ± 9.13	35.31 ± 6.67	**<0.001**
Decreased skeletal muscle mass				**<0.001**
Yes	13 (38.24)	23 (82.14)	42 (95.45)	
No	21 (61.76)	5 (17.86)	2 (4.55)	
Visceral obesity				0.171
Yes	4 (11.76)	4 (14.29)	12 (27.27)	
No	30 (88.24)	24 (85.71)	32 (72.73)	

Values are presented as N (%), the mean ± SD, or the median (interquartile range). BMI, body mass index; ESR, erythrocyte sedimentation rate; CRP, C-reactive protein; Alb, albumin; SAI, subcutaneous adipose index; VAI, visceral adipose index; TAI, total adipose index; SMI, skeletal muscle index. Bold values indicate significant differences (*p*<0.05).

**Figure 3:**
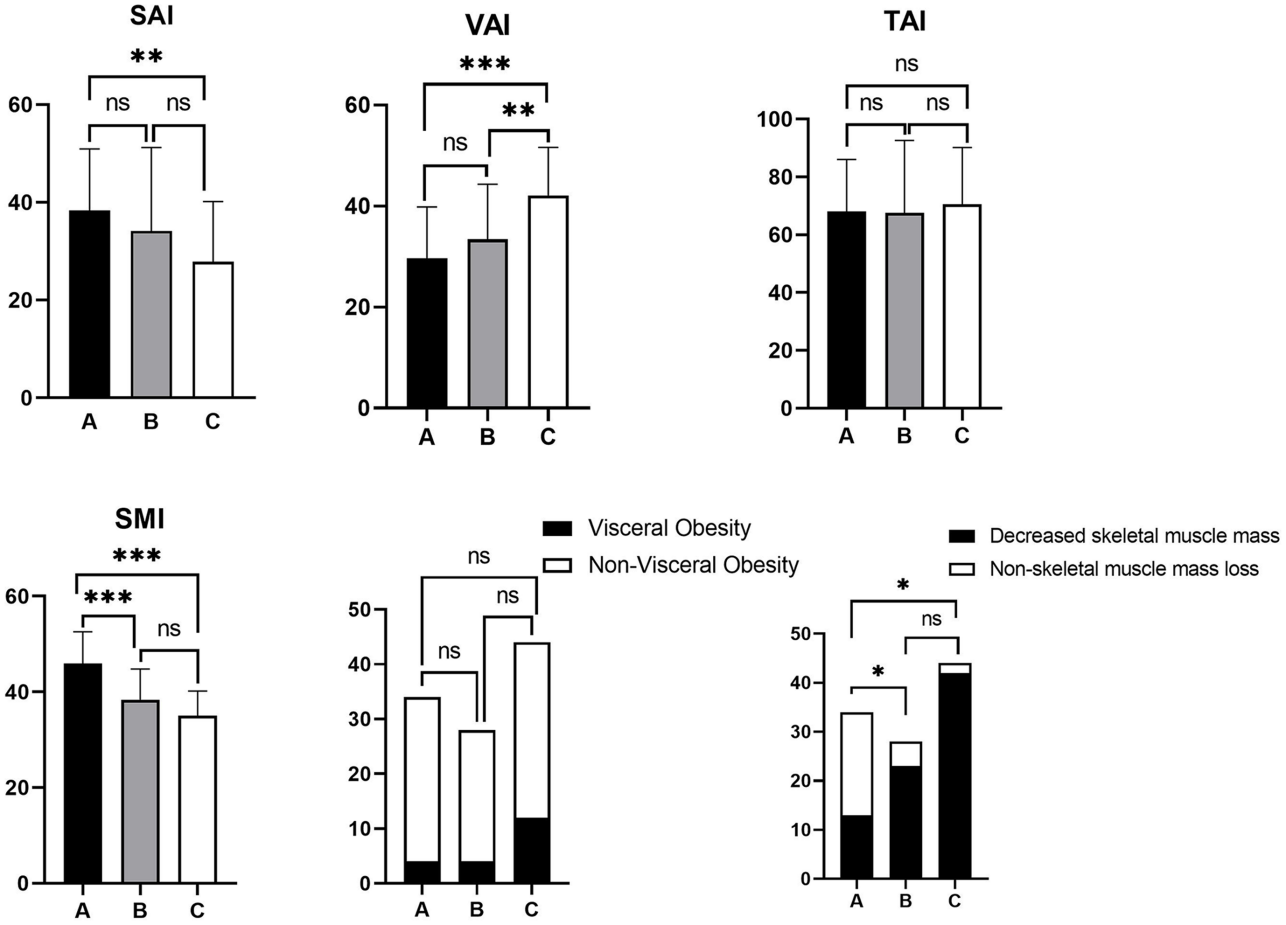
Histogram of multiple comparisons of activity status between groups. A: disease remission; B: mild activity; C: moderate-to-severe disease activity. SAI, subcutaneous adipose index; VAI, visceral adipose index; TAI, total adipose index; SMI, skeletal muscle index. ***p < 0.001, **p < 0.01, *p < 0.05, ^ns^*p* > 0.05 (not significant).

The differences in SAI (*p* = 0.010), SMI (*p* < 0.001), VAI (*p* < 0.001) and decreased skeletal muscle mass (*p* < 0.001) were statistically significant between the different activity statuses. After multiple comparisons between groups, the differences between the four body composition parameters mentioned above (SAI: *p* = 0.008, VAI: *p* < 0.001, SMI: *p* < 0.001, and decreased skeletal muscle mass: *p* < 0.05) were statistically significant between patients in remission and moderate-to-severe activity, whereas when comparing body composition between patients in remission and mild activity, only SMI (*p* = 0.002) and decreased skeletal muscle mass (*p* < 0.05) showed significant differences. Similarly, a significant difference in VAI (*p* = 0.009) was found only between patients with mildly and moderately active. TAI and visceral obesity showed no significant difference between the three groups and when compared between the two groups (*p* > 0.05) (Table 2; Figure 3).

### 3.4. Differential analysis of body composition parameters of CD patients In different disease behavior groups

**Figure 4:**
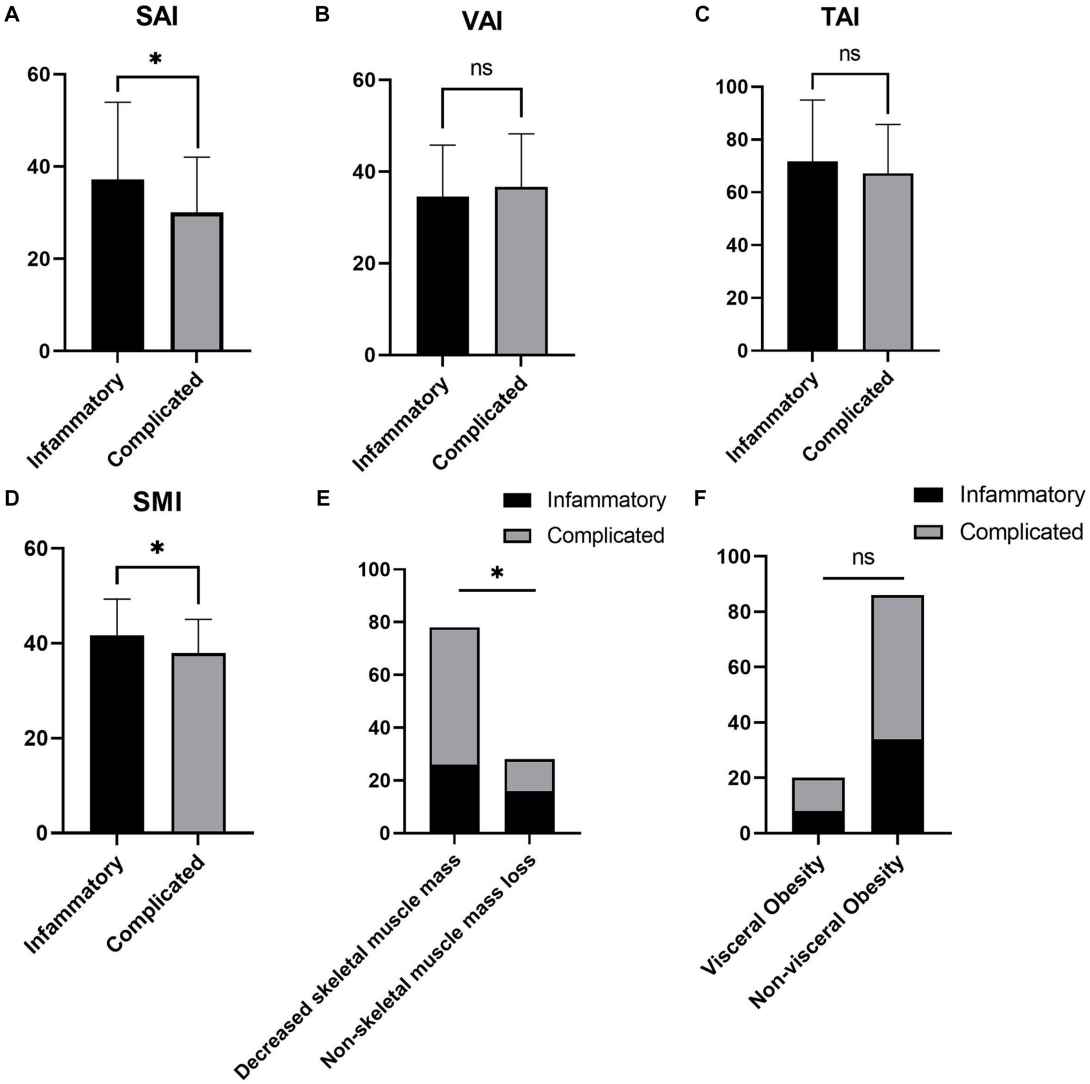
Comparison of body composition parameters between the inflammatory and complex behavior groups. **(A–F)** represent the differential analysis of SAI **(A)**, VAI **(B)**, TAI **(C)**, SMI **(D)**, decreased skeletal muscle mass **(E)**, and visceral obesity **(F)** between the two groups, respectively. SAI, subcutaneous adipose index; VAI, visceral adipose index; TAI, total adipose index; SMI, skeletal muscle index. *p < 0.05, ^ns^*p* > 0.05 (not significant).

There were 42 (39.62%) and 64 (60.38%) patients in the inflammatory and complex behavior groups, respectively, with no significant difference in demographics between the two groups (*p* > 0.05). ESR (*p* = 0.045) and CRP (*p* = 0.005) were higher in the complex behavior group. SAI (*p* = 0.015), SMI (*p* = 0.011) and decreased skeletal muscle mass (*p* = 0.027) were significantly different between the two groups. The complex behavior group had a lower SAI and SMI and a higher prevalence of decreased skeletal muscle mass. The differences in BMI (*p* = 0.090), VAI (*p* = 0.347), TAI (*p* = 0.273) and visceral obesity (*p* = 0.969) were not significant between the two groups (Supplementary Table S2; Figure 4).

### 3.5. Analysis of independent correlates of disease severity

**Figure 5:**
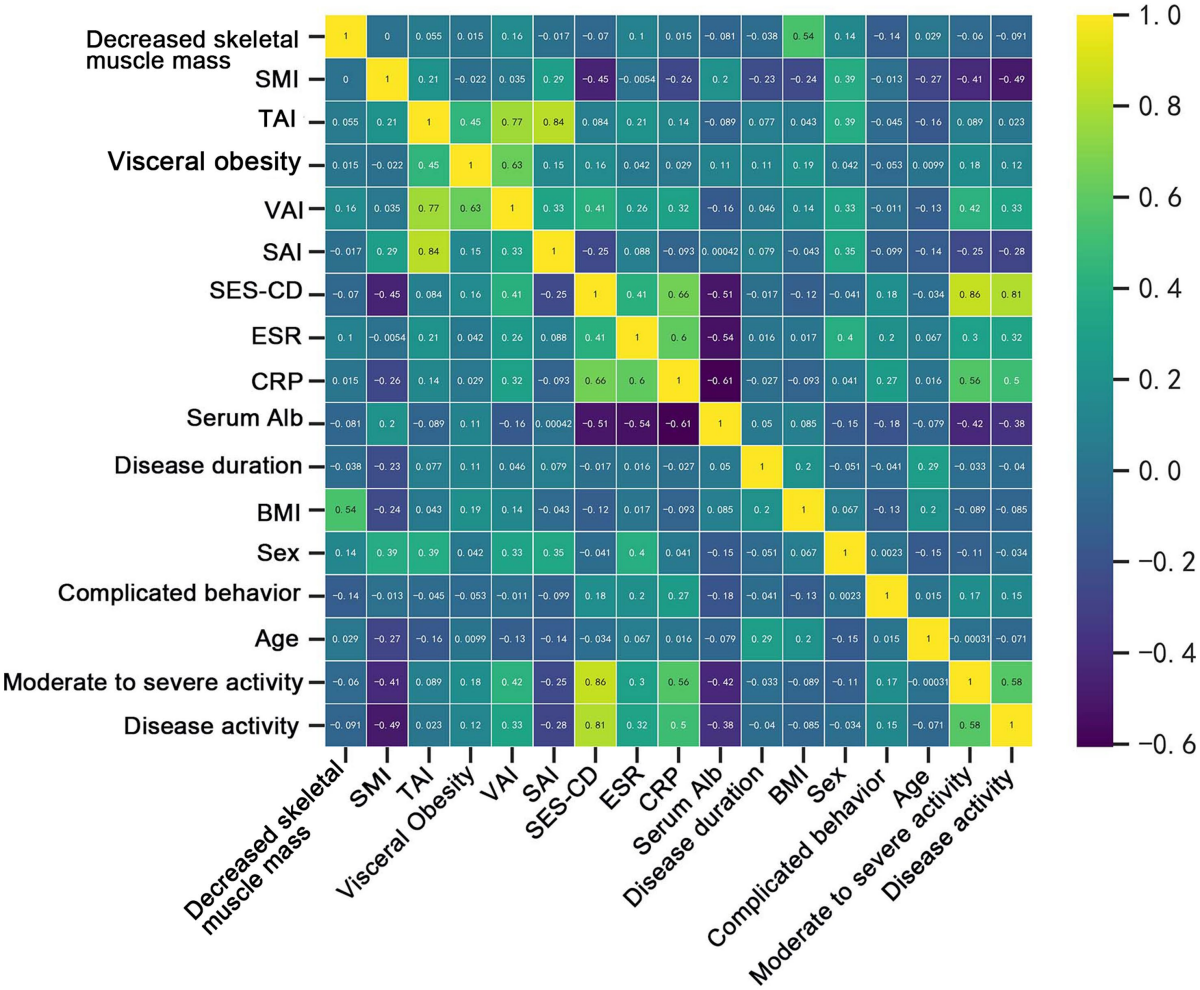
Matrix of correlation coefficients between body composition parameters, laboratory data, and outcome variables.

**Figure 6:**
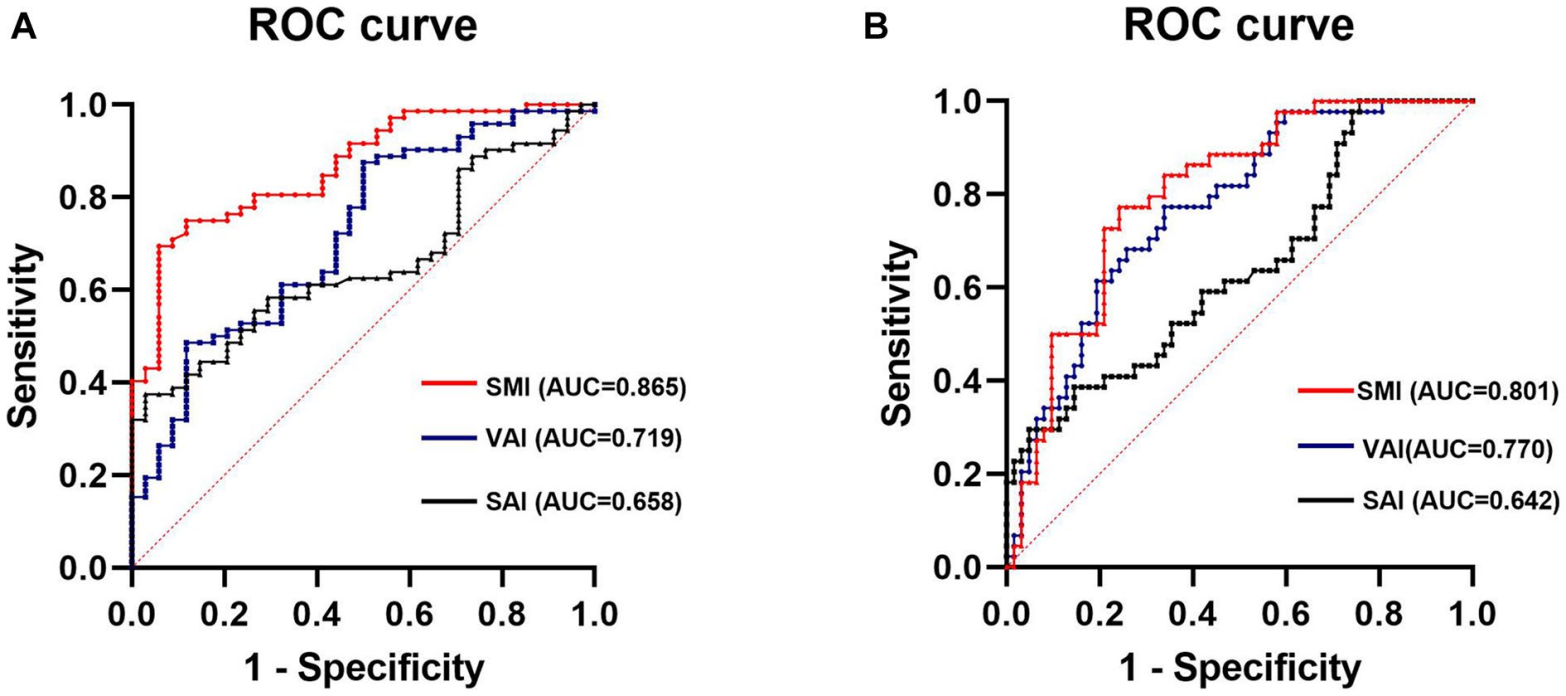
ROC analysis of SMI, VAI, and SAI diagnosed disease activity **(A)** and moderate-to-severe activity **(B)**. SMI, skeletal muscle index; VAI, visceral adipose index; SAI, subcutaneous adipose index.

The results of the Spearman correlation analysis showed correlations between several body composition parameters (SMI, SAI, VAI) and laboratory data (ESR and VAI: *p* = 0.006; CRP and SMI: *p* = 0.008, CRP and VAI: *p* = 0.001; albumin and SMI: *p* = 0.041), as well as disease activity status (disease activity and SMI: *p* < 0.001, SAI: *p* = 0.004, VAI: *p* < 0.001; moderate-to-severe activity and SMI: *p* < 0.001, SAI: *p* = 0.011, VAI: *p* < 0.001) (Figure 5; Supplementary Table S3). In order to understand the independent effects of disease activity, we categorized patients into a remission group and an activity group based on their disease activity status, with the activity group including patients with both mild and moderate-to-severe disease activity. Additionally, we divided patients into non-moderate-to-severe activity group and moderate-to-severe activity group according to the degree of disease activity, with the non-moderate-to-severe activity group including patients in remission and patients with mild disease activity. The above variables were included in a multivariate binary logistic regression analysis and adjusted for the effect of the gender covariate, the results showed that SAI (*p* = 0.006 and *p* = 0.001), VAI (*p* < 0.001 and *p* < 0.001) and SMI (*p* < 0.001 and *p* = 0.007) were independently associated with disease activity (including mild and moderate-to-severe activity) and moderate-to-severe activity, respectively (Table 3). In addition, considering the certain correlation between ESR and CRP (Figure 5; Supplementary Table S3), we included both variables separately in the multivariate analysis. The results still demonstrated that SAI, VAI, and SMI were independent factors associated with the degree of disease activity (Supplementary Tables S4, S5). ROC analysis showed that SAI, VAI and SMI were all effective in diagnosing disease activity status and identifying moderate-to-severe disease activity level, with AUC values all greater than 0.65. Among them, SMI performs best in diagnosing disease activity and moderate-to-severe activity, with AUC values of 0.865 and 0.801, respectively (Figure 6).

**Table 3:** Multivariate analysis of disease activity and moderate-to-severe activity.

	Disease activity status			Moderate-to-severe disease activity		
	OR	95%CI	*p* value	OR	95%CI	*p* value
SAI	0.92	(0.87–0.98)	**0.006**	0.88	(0.82–0.95)	**0.001**
VAI	1.15	(1.07–1.23)	<**0.001**	1.24	(1.11–1.38)	<**0.001**
SMI	0.82	(0.73–0.91)	<**0.001**	0.86	(0.78–0.96)	**0.007**
Serum Alb (g/l)	0.85	(0.67–1.07)	0.169	0.82	(0.66–1.01)	0.061
CRP (mg/l)	1.19	(0.99–1.44)	0.070	1.02	(0.97–1.08)	0.426
ESR (mm/h)	1.03	(0.97–1.10)	0.344	0.99	(0.94–1.05)	0.713

SAI, subcutaneous adipose index; VAI, visceral adipose index; SMI, skeletal muscle index; Alb, albumin; CRP, C-reactive protein; ESR, erythrocyte sedimentation rate. Bold values indicate significant differences (*p*<0.05).

Binary logistic regression analysis was used to explore independent correlates of complex disease behavior. Variables that were statistically significant in the univariate analysis (SAI: *p* = 0.016, SMI: *p* = 0.015, and decreased skeletal muscle mass: *p* = 0.030) were included in the multivariate analysis. However, the results indicated that none of these variables were independent correlates of complex disease behavior (SAI: *p* = 0.065, SMI: *p* = 0.139, and decreased skeletal muscle mass: *p* = 0.761) (Table 4). This is consistent with the results of the Spearman correlation analysis (Figure 5), which showed no significant correlation between complex disease behavior and body composition parameters.

**Table 4:** Univariate and multivariate analyses of complex disease behavior.

	Univariate analysis				Multivariate analysis	
	OR	95%CI	*p* value	OR	95%CI	*p* value
Age (years)	0.99	(0.95–1.04)	0.753			
Sex	1.01	(0.45–2.27)	0.981			
Disease duration (years)	0.99	(0.86–1.14)	0.843			
BMI (kg/m^2^)	0.90	(0.80–1.01)	0.095			
SAI (cm^2^/m^2^)	0.97	(0.94–0.99)	**0.016**	0.97	(0.94–1.00)	0.065
VAI (cm^2^/m^2^)	1.02	(0.98–1.05)	0.344			
TAI (cm^2^/m^2^)	0.99	(0.97–1.01)	0.271			
SMI (cm^2^/m^2^)	0.93	(0.88–0.99)	**0.015**	0.95	(0.88–1.02)	0.139
Visceral obesity	0.98	(0.36–2.65)	0.969			
Decreased skeletal muscle mass	2.67	(1.10–6.46)	**0.030**	1.20	(0.37–3.83)	0.761

BMI, body mass index; SAI, subcutaneous adipose index; VAI, visceral adipose index; TAI, total adipose index; SMI, skeletal muscle index.

## 4. Discussion

The results of this study showed a high prevalence of decreased skeletal muscle mass in patients with CD (75%), which is consistent with the results of our recently published systematic review (30). This systematic review included 39 studies for analysis, and more than half of them reported a prevalence of sarcopenia >50% in patients with CD. Additionally, according to the Global Leadership Initiative on Malnutrition (GLIM) (31), which proposes the inclusion of reduced muscle mass as one of the diagnostic criteria for malnutrition in adults, almost 2/3 of patients in our study cohort were malnourished, which was significantly higher than the prevalence of malnutrition defined by BMI (26.42%), and there were significantly more viscerally obese patients than overweight patients defined by BMI (18.87% vs. 11.32%). This suggests that body composition-related parameters may be a more sensitive indicator than BMI to reflect the nutritional status of patients.

Body composition can reflect the severity of disease by characterizing the nutritional status of CD patients. The results of this study showed that body composition changes (SMI, SAI, VAI and decreased skeletal muscle mass) in CD patients differed between disease activity states, and multivariate analysis indicated that disease activity states were independently and negatively correlated with SMI and SAI and independently and positively correlated with VAI. The proliferation of mesenteric adipose tissue around the lesion, forming “crawling fat,” is considered an important feature of CD (32). Various inflammatory factors, such as TNF-α, chemokines and resistin, secreted by proliferating mesenteric adipose tissue play an important role in the progression of disease inflammation (33). Since peri-intestinal adipose tissue is an important component of VAT, it shows a positive correlation with disease activity status. While SAT and VAT have different metabolic profiles, SAT has been shown to play a protective role in the metabolism of prostate cancer patients (34). In CD patients, similar characteristics seem to exist, as the disease activity increases, patients’ SAT catabolism decreases and protective metabolic effects diminish, thus keeping TAI in dynamic balance, which also provides a reasonable explanation for the lack of a significant correlation between TAI and disease severity. At the same time, we found that SMI performed best in reflecting the disease activity status. The current “gut-muscle axis” hypothesis, supported by most evidence, suggests that the gut microbiota shifts to a proinflammatory role, altering host metabolic and immune responses, maintaining low levels of inflammation, and upregulating several molecular pathways associated with reduced muscle mass (7, 35, 36), such that the more severe the disease in CD patients, the lower the SMI. The results of this study are consistent with the findings of Labarthe et al. (37), who analyzed the correlation between body composition and disease activity level in CD patients using MRI images, although the disease activity level score (Harvey-Bradshaw) used by this team was defined according to the patients’ clinical symptoms. Similarly, the results of a recent study of 71 CD patients with activity follow-up found a negative correlation between SAI and SMI and the disease activity level (38), which is similar to our findings. In addition, we found more significant differences in body composition-related parameters when the activity levels differed more (remission vs. moderate-to-severe activity). This may be because the inflammatory level of the disease tends to fluctuate, and the level of disease activity is more likely to change between adjacent subgroups, whereas the distribution of body composition changes relatively slowly and thus some of the body composition parameters do not differ significantly between adjacent subgroups.

Our study reported a strong association between body composition parameters and disease activity status, but two other studies (8, 39) did not find such an association. We concluded that this discrepancy may be attributed to several reasons. Firstly, both of these studies used the Crohn’s disease activity index (CDAI) scoring system to assess disease activity, which depends mainly on subjective sensations such as the degree of abdominal pain in patients, and pain thresholds vary from patient to patient, which may affect the accurate assessment of disease activity (40). In contrast, the endoscopic scoring system used in this study provides a more objective picture of bowel inflammation and allows patients to report disease activity status more consistently between visits or between clinicians (41). Secondly, previous studies did not differentiate between patients based on their treatment methods. Considering that different drug treatments may influence body composition distribution, we only included patients who treated with IFX. Shen et al. (17) showed that increased visceral adiposity was associated with attenuated mucosal healing in IFX-treated patients, which is consistent with the findings of our study. Lastly, differences in body composition distribution may be influenced by race (42), and differences in lifestyle and dietary habits exist between races, which may also affect the consistency of results between studies.

Nonetheless, our results showed that SAI and SMI were lower and the prevalence of decreased skeletal muscle mass was higher in CD patients with complex disease behavior, and although the group differences in VAT were not significant, VAT still tended to be higher in the complex disease behavior group. Erhayiem et al. (43) showed that CD patients with higher VAT were more likely to have complex disease behavior after body composition analysis of CT images of 50 CD patients, which was similarly concluded in a subsequent study (44–48). Zhou et al. (49), by retrospectively analyzing body composition in 122 CD patients, found that a lower SMI may be an indicator of complex disease behavior. However, although our study revealed variability in some body composition parameters among CD patients with different disease behaviors, it was not their independent correlate, which is consistent with the results of a recent study (50). This may be because intestinal complications such as stricture or penetration are a further progression of poorly controlled disease activity and are influenced by a combination of factors such as altered body composition, poor drug therapy and an active intestinal inflammatory response; therefore, altered body composition is not an independent correlate of complex disease behavior.

Notably, this study also found a correlation between some body composition parameters and inflammatory indicators. Levels of inflammatory indicators (ESR and CRP) are elevated in patients with active disease or with complex disease behavior, which further supports the conclusion that body composition can reflect disease severity. However, inflammatory markers can be influenced by infectious diseases or medication use at other sites and are therefore less specific in reflecting disease severity in CD patients. In contrast, our study found that SAI, VAI, and SMI were independent correlates of disease activity status and moderate-to-severe disease activity level after correcting for inflammatory indicators in a multivariate analysis. This suggests that body composition parameters are more advantageous than inflammatory indicators in assessing the course of disease activity. Therefore, monitoring changes in body composition in CD patients and early increasing the dosage of IFX or adopting interventions such as nutritional management and physical exercise for patients with a tendency of increased visceral fat and decreased skeletal muscle mass may help prevent further disease progression and allow patients to maintain a state of long-term disease remission (51).

There are numerous methods for measuring body composition, including dual-energy X-ray absorptiometry (DXA), bioelectrical impedance analysis (BIA), CT, and MRI (5). Among them, DXA and BIA show low performance in quantifying the specific distribution of body composition, while CT and MRI provide high resolution of soft tissues and are considered the gold standard for body composition assessment (52, 53). The QCT used in this study is an emerging quantitative technique mainly applied to body composition and bone density measurements, which is consistent with the effects of body composition measurement tools commonly used in previous scientific studies; moreover, QCT can easily, accurately and automatically calculate patients’ body composition information without the burden of additional radiation and cost by using patients’ regularly reviewed CT data (54). Thus, QCT can help clinics to easily and efficiently monitor patients’ body composition information.

This study has several limitations. First, it was a single-center, retrospective investigation and was based on a single-race population analysis only. The distribution of body composition may be influenced by race, and therefore, the present results still need to be evaluated in a multicenter, prospective, multirace population validation study. Second, the limited sample size of the study and the uneven proportion of people in different disease activity subgroups and disease behavior subgroups may have caused some bias in the study results. Finally, only patients treated with IFX were included in this study. Therefore, in our future studies, we plan to further include patients treated with different drugs and to expand the sample size of the study to better control for potential confounders.

## 5. Conclusion

In conclusion, this study shows that there is a correlation between body composition parameters and disease severity in patients with CD. Patients with a higher VAT and a lower SAT and SMI may have higher disease activity and more complex disease behavior, and SMI can be a reliable indicator of disease activity status. Regular monitoring of patients’ body composition trends by QCT may allow early identification of the disease severity process and enable the clinical use of timely targeted treatment or management measures to promote a good treatment outcome and prognosis.

## Data availability statement

The raw data supporting the conclusions of this article will be made available by the authors, without undue reservation.

## Ethics statement

The studies involving humans were approved by the Medical Ethics Committee of Chongqing General Hospital. The studies were conducted in accordance with the local legislation and institutional requirements. Written informed consent for participation was not required for this study in accordance with the national legislation and the institutional requirements.

## Author contributions

WuT: conceptualization and writing—original draft preparation. JL and LY: software. JD: validation. WuT and GX: formal analysis. RY and WeT: investigation. KL: resources. WeT and LZ: data curation. GX and KL: writing—review and editing. YZ: visualization. WuT and KL: funding acquisition. All authors contributed to the article and approved the submitted version.

## Funding

This research was funded by the Chongqing Science and Technology Bureau of Science and Technology Innovation and Application Development Special Project (Grant No. cstc2020jscx-sbqwX0015) and Chongqing Postgraduate Research Innovation Project (Grant No. CYS22391).

## Conflict of interest

The authors declare that the research was conducted in the absence of any commercial or financial relationships that could be construed as a potential conflict of interest.

## Publisher’s note

All claims expressed in this article are solely those of the authors and do not necessarily represent those of their affiliated organizations, or those of the publisher, the editors and the reviewers. Any product that may be evaluated in this article, or claim that may be made by its manufacturer, is not guaranteed or endorsed by the publisher.

## References

[1] Palmese F Del Toro R Di Marzio G Cataleta P Sama MG Domenicali M. Sarcopenia and vitamin d deficiency in patients with crohn’s disease: pathological conditions that should be linked together. Nutrients. (2021) 13:1378. doi: 10.3390/nu13041378, PMID: 33923948 PMC8074054

[2] Scaldaferri F Pizzoferrato M Lopetuso LR Musca T Ingravalle F Sicignano LL. Nutrition and IBD: malnutrition and/or sarcopenia? A practical guide. Gastroenterol Res Pract. (2017) 2017:8646495. doi: 10.1155/2017/8646495, PMID: 28127306 PMC5239980

[3] Massironi S Vigano C Palermo A Pirola L Mulinacci G Allocca M. Inflammation and malnutrition in inflammatory bowel disease. Lancet Gastroenterol Hepatol. (2023) 8:579–90. doi: 10.1016/S2468-1253(23)00011-0, PMID: 36933563

[4] Ananthakrishnan AN. Frailty in patients with inflammatory bowel disease. Gastroenterol Hepatol (N Y). (2021) 17:263–8. PMID: 34776800 PMC8576843

[5] Ding NS Tassone D Al Bakir I Wu K Thompson AJ Connell WR. Systematic review: the impact and importance of body composition in inflammatory bowel disease. J Crohn’s Colitis. (2022) 16:1475–92. doi: 10.1093/ecco-jcc/jjac041, PMID: 35325076 PMC9455788

[6] Ha C Martin A Sepich-Poore GD Shi B Wang Y Gouin K. Translocation of viable gut microbiota to mesenteric adipose drives formation of creeping fat in humans. Cells. (2020) 183:666–683.e17. doi: 10.1016/j.cell.2020.09.009, PMID: 32991841 PMC7521382

[7] Dhaliwal A Quinlan JI Overthrow K Greig C Lord JM Armstrong MJ. Sarcopenia in inflammatory bowel disease: a narrative overview. Nutrients. (2021) 13:656. doi: 10.3390/nu13020656, PMID: 33671473 PMC7922969

[8] Boparai G Kedia S Kandasamy D Sharma R Madhusudhan KS Dash NR. Combination of sarcopenia and high visceral fat predict poor outcomes in patients with Crohn’s disease. Eur J Clin Nutr. (2021) 75:1491–8. doi: 10.1038/s41430-021-00857-x 33531636

[9] Bamba S Inatomi O Takahashi K Morita Y Imai T Ohno M. Assessment of body composition from CT images at the level of the third lumbar vertebra in inflammatory bowel disease. Inflamm Bowel Dis. (2021) 27:1435–42. doi: 10.1093/ibd/izaa306, PMID: 33236765

[10] Yasueda A Sekido Y Takeda T Ogino T Miyoshi N Takahashi H. Sarcopenia hinders the decline in disease activity after surgery for people with Crohn’s disease: preliminary results. Nutrition. (2022) 94:111526. doi: 10.1016/j.nut.2021.111526, PMID: 34861460

[11] Galata C Hodapp J Weiß C Karampinis I Vassilev G Reißfelder C. Skeletal muscle mass index predicts postoperative complications in intestinal surgery for crohn’s disease. Jpen-Parenter Enter. (2020) 44:714–21. doi: 10.1002/jpen.1696, PMID: 31444789

[12] Nardone OM Ponsiglione A de Sire R Calabrese G Liuzzi R Testa A. Impact of sarcopenia on clinical outcomes in a cohort of Caucasian active Crohn’s disease patients undergoing multidetector CT-Enterography. Nutrients. (2022) 14:3460. doi: 10.3390/nu14173460, PMID: 36079718 PMC9458031

[13] Ando K Uehara K Sugiyama Y Kobayashi Y Murakami Y Sato H. Correlation among body composition parameters and Long-term outcomes in crohn’s disease after anti-TNF therapy. Front Nutr. (2022) 9:9. doi: 10.3389/fnut.2022.765209, PMID: 35433773 PMC9010511

[14] Parmentier-Decrucq E Duhamel A Ernst O Fermont C Louvet A Vernier-Massouille G. Effects of infliximab therapy on abdominal fat and metabolic profile in patients with Crohnʼs disease. Inflamm Bowel Dis. (2009) 15:1476–84. doi: 10.1002/ibd.20931, PMID: 19291781

[15] Zhang Z Yu X Fang N Long X Ruan X Qiu J. Can visceral adipose tissue and skeletal muscle predict recurrence of newly diagnosed Crohn’s disease in different treatments. BMC Gastroenterol. (2022) 22:250. doi: 10.1186/s12876-022-02327-5, PMID: 35585617 PMC9116006

[16] Lim Z Welman CJ Raymond W Thin L. The effect of adiposity on anti-tumor necrosis factor-alpha levels and loss of response in crohn’s disease patients. Clin Transl Gastroenterol. (2020) 11:e233. doi: 10.14309/ctg.0000000000000233, PMID: 33094963 PMC7515616

[17] Shen W Cao L Li Y Cai X Ge Y Zhu W. Visceral fat is associated with mucosal healing of infliximab treatment in crohn’s disease. Dis Colon Rectum. (2018) 61:706–12. doi: 10.1097/DCR.0000000000001074, PMID: 29722729

[18] Gajendran M Loganathan P Catinella AP Hashash JG. A comprehensive review and update on Crohn’s disease. Dis Mon. (2018) 64:20–57. doi: 10.1016/j.disamonth.2017.07.001 28826742

[19] Lichtenstein GR Loftus EV Isaacs KL Regueiro MD Gerson LB Sands BE. ACG clinical guideline: management of crohn’s disease in adults. Am J Gastroenterol. (2018) 113:481–517. doi: 10.1038/ajg.2018.27, PMID: 29610508

[20] Van Assche G Dignass A Panes J Beaugerie L Karagiannis J Allez M. The second European evidence-based consensus on the diagnosis and management of Crohn’s disease: definitions and diagnosis. J Crohns Colitis. (2010) 4:7–27. doi: 10.1016/j.crohns.2009.12.003, PMID: 21122488

[21] Satsangi J Silverberg MS Vermeire S Colombel JF. The Montreal classification of inflammatory bowel disease: controversies, consensus, and implications. Gut. (2006) 55:749–53. doi: 10.1136/gut.2005.082909, PMID: 16698746 PMC1856208

[22] Gravina AG Pellegrino R Romeo M Palladino G Cipullo M Iadanza G. Quality of bowel preparation in patients with inflammatory bowel disease undergoing colonoscopy: what factors to consider? World J Gastrointest Endosc. (2023) 15:133–45. doi: 10.4253/wjge.v15.i3.133, PMID: 37034970 PMC10080552

[23] Khanna R Zou G D’Haens G Rutgeerts P McDonald JWD Daperno M. Reliability among central readers in the evaluation of endoscopic findings from patients with Crohn’s disease. Gut. (2016) 65:1119–25. doi: 10.1136/gutjnl-2014-308973, PMID: 25935574

[24] Khanna R Zou G Stitt L Feagan BG Sandborn WJ Rutgeerts P. Responsiveness of endoscopic indices of disease activity for crohn’s disease. Am J Gastroenterol. (2017) 112:1584–92. doi: 10.1038/ajg.2016.580 28071654

[25] Sipponen T Nuutinen H Turunen U Farkkila M. Endoscopic evaluation of Crohn’s disease activity: comparison of the CDEIS and the SES-CD. Inflamm Bowel Dis. (2010) 16:2131–6. doi: 10.1002/ibd.21300 20848462

[26] Gomez-Perez SL Haus JM Sheean P Patel B Mar W Chaudhry V. Measuring abdominal circumference and skeletal muscle from a single cross-sectional computed tomography image: a step-by-step guide for clinicians using national institutes of health ImageJ. JPEN J Parenter Enteral Nutr. (2016) 40:308–18. doi: 10.1177/0148607115604149, PMID: 26392166 PMC4767633

[27] Bani HE Demontiero O Vogrin S Ng A Duque G. Marrow adipose tissue in older men: association with visceral and subcutaneous fat, bone volume, metabolism, and inflammation. Calcif Tissue Int. (2018) 103:164–74. doi: 10.1007/s00223-018-0412-6, PMID: 29582133

[28] Prado CM Lieffers JR McCargar LJ Reiman T Sawyer MB Martin L. Prevalence and clinical implications of sarcopenic obesity in patients with solid tumours of the respiratory and gastrointestinal tracts: a population-based study. Lancet Oncol. (2008) 9:629–35. doi: 10.1016/S1470-2045(08)70153-0, PMID: 18539529

[29] Despres JP Lamarche B. Effects of diet and physical activity on adiposity and body fat distribution: implications for the prevention of cardiovascular disease. Nutr Res Rev. (1993) 6:137–59. doi: 10.1079/NRR19930010, PMID: 19094306

[30] Tang W Xie G Wang D Li T Ren Y Li J. Imaging-based assessment of body composition in patients with Crohn’s disease: a systematic review. Int J Color Dis. (2023) 38:126. doi: 10.1007/s00384-023-04413-w, PMID: 37171498

[31] Cederholm T Jensen GL Correia M Gonzalez MC Fukushima R Higashiguchi T. GLIM criteria for the diagnosis of malnutrition - a consensus report from the global clinical nutrition community. Clin Nutr. (2019) 38:1–9. doi: 10.1016/j.clnu.2018.08.002, PMID: 30181091

[32] Rivera ED Coffey JC Walsh D Ehrenpreis ED. The mesentery, systemic inflammation, and crohn’s disease. Inflamm Bowel Dis. (2019) 25:226–34. doi: 10.1093/ibd/izy201, PMID: 29920595

[33] Karaskova E Velganova-Veghova M Geryk M Foltenova H Kucerova V Karasek D. Role of adipose tissue in inflammatory bowel disease. Int J Mol Sci. (2021) 22:22. doi: 10.3390/ijms22084226, PMID: 33921758 PMC8073530

[34] Antoun S Bayar A Ileana E Laplanche A Fizazi K di Palma M. High subcutaneous adipose tissue predicts the prognosis in metastatic castration-resistant prostate cancer patients in post chemotherapy setting. Eur J Cancer. (2015) 51:2570–7. doi: 10.1016/j.ejca.2015.07.042, PMID: 26278649

[35] Ehlers L Bannert K Rohde S Berlin P Reiner J Wiese M. Preclinical insights into the gut-skeletal muscle axis in chronic gastrointestinal diseases. J Cell Mol Med. (2020) 24:8304–14. doi: 10.1111/jcmm.15554, PMID: 32628812 PMC7412689

[36] Marco P Roberto DS Fabio I Maria CM Valentina P Anna MM. Characterization of sarcopenia in an IBD population attending an italian gastroenterology tertiary center. Nutrients. (2019) 11:2281. doi: 10.3390/nu11102281 31554166 PMC6835412

[37] Labarthe G Dolores M Verdalle-Cazes M Charpentier C Roullee P Dacher JN. Magnetic resonance imaging assessment of body composition parameters in Crohn’s disease. Dig Liver Dis. (2020) 52:878–84. doi: 10.1016/j.dld.2020.06.024, PMID: 32622612

[38] Lee JY Kim KW Ko Y Oh CH Kim BH Park SJ. Serial changes in body composition and the association with disease activity during treatment in patients with Crohn’s disease. Diagnostics. (2022) 12:2804. doi: 10.3390/diagnostics12112804, PMID: 36428862 PMC9689369

[39] Yadav DP Kedia S Madhusudhan KS Bopanna S Goyal S Jain S. Body composition in crohn’s disease and ulcerative colitis: correlation with disease severity and duration. Can J Gastroenterol. (2017) 2017:1–8. doi: 10.1155/2017/1215035, PMID: 29226115 PMC5684551

[40] Sostegni R Daperno M Scaglione N Lavagna A Rocca R Pera A. Review article: Crohn’s disease: monitoring disease activity. Aliment Pharmacol Ther. (2003) 17:11–7. doi: 10.1046/j.1365-2036.17.s2.17.x 12786607

[41] Panes J Jairath V Levesque BG. Advances in use of endoscopy, radiology, and biomarkers to monitor inflammatory bowel diseases. Gastroenterology. (2017) 152:362–373.e3. doi: 10.1053/j.gastro.2016.10.005 27751880

[42] Argeny S Tamandl D Scharitzer M Stift A Bergmann M Riss S. Visceral fat area measured with computed tomography does not predict postoperative course in Crohn s disease patients. PLoS One. (2018) 13:e202220. doi: 10.1371/journal.pone.0202220, PMID: 30133500 PMC6104989

[43] Erhayiem B Dhingsa R Hawkey CJ Subramanian V. Ratio of visceral to subcutaneous fat area is a biomarker of complicated Crohn’s disease. Clin Gastroenterol Hepatol. (2011) 9:684–687.e1. doi: 10.1016/j.cgh.2011.05.005, PMID: 21642015

[44] Kurban M Zeng N Wang M Liu H Wu J Feng G. Role of human body composition analysis and malnutrition risk questionnaire in the assessment of nutritional status of patients with initially diagnosed crohn’s disease. Front Med. (2020) 7:7. doi: 10.3389/fmed.2020.00106, PMID: 32328493 PMC7161551

[45] Velho S Morão B Gouveia C Agostinho L Torres J Maio R. Body composition and Crohn’s disease behavior: is adiposity the main game changer? Nutrition. (2023) 108:111959. doi: 10.1016/j.nut.2022.111959, PMID: 36709640

[46] Bryant RV Schultz CG Ooi S Goess C Costello SP Vincent AD. Visceral adipose tissue is associated with stricturing crohn’s disease behavior, fecal calprotectin, and quality of life. Inflamm Bowel Dis. (2019) 25:592–600. doi: 10.1093/ibd/izy278, PMID: 30215805

[47] Büning C von Kraft C Hermsdorf M Gentz E Wirth EK Valentini L. Visceral adipose tissue in patients with crohnʼs disease correlates with disease activity, inflammatory markers, and outcome. Inflamm Bowel Dis. (2015) 21:2590–7. doi: 10.1097/MIB.0000000000000527, PMID: 26222339

[48] Yuan G He Y Cao Q Tang M Xie Z Qiu Y. Visceral adipose volume is correlated with surgical tissue fibrosis in Crohn’s disease of the small bowel. Gastroenterol Rep. (2022) 10:10. doi: 10.1093/gastro/goac044, PMID: 36042948 PMC9420045

[49] Zhou Z Xiong Z Xie Q Xiao P Zhang Q Gu J. Computed tomography-based multiple body composition parameters predict outcomes in Crohn’s disease. Insights Imaging. (2021) 12:135. doi: 10.1186/s13244-021-01083-6, PMID: 34564786 PMC8464641

[50] Barajas Ordonez F Melekh B Rodríguez-Feria P Melekh O Thormann M Damm R. Body composition predictors of complicated Crohn’s disease. Dig Dis. (2023) 41:589–99. doi: 10.1159/000529426, PMID: 36720207 PMC10777712

[51] Nishikawa H Nakamura S Miyazaki T Kakimoto K Fukunishi S Asai A. Inflammatory bowel disease and sarcopenia: its mechanism and clinical importance. J Clin Med. (2021) 10:10. doi: 10.3390/jcm10184214, PMID: 34575326 PMC8470813

[52] Kullberg J Brandberg J Angelhed J Frimmel H Bergelin E Strid L. Whole-body adipose tissue analysis: comparison of MRI, CT and dual energy X-ray absorptiometry. Br J Radiol. (2009) 82:123–30. doi: 10.1259/bjr/80083156, PMID: 19168691

[53] Cruz-Jentoft AJ Baeyens JP Bauer JM Boirie Y Cederholm T Landi F. Sarcopenia: European consensus on definition and diagnosis: report of the European working group on sarcopenia in older people. Age Ageing. (2010) 39:412–23. doi: 10.1093/ageing/afq034, PMID: 20392703 PMC2886201

[54] Sheu Y Marshall LM Holton KF Caserotti P Boudreau RM Strotmeyer ES. Abdominal body composition measured by quantitative computed tomography and risk of non-spine fractures: the osteoporotic fractures in men (MrOS) study. Osteoporos Int. (2013) 24:2231–41. doi: 10.1007/s00198-013-2322-9, PMID: 23471565 PMC3947542

