# Supplementary material for: Body composition parameters correlate with the endoscopic severity in Crohn’s disease patients treated with infliximab
Source: Front Nutr. 2023 Aug 21;10:1251448. doi: 10.3389/fnut.2023.1251448 (PMC10478258; doi:10.3389/fnut.2023.1251448)
Supplement: Supplementary file 1 [file Data_Sheet_1.docx]

Supplementary Material

**Table S1. Consistency analysis of body composition calculated by QCT pro and ImageJ.**

|  | ICC | 95%CI |
| --- | --- | --- |
| SFA | 0.970 | (0.956, 0.979) |
| VFA | 0.988 | (0.983, 0.992) |
| SMA | 0.985 | (0.979, 0.990) |

ICC, intraclass correlation coefficient; SFA, subcutaneous fat area; VFA, visceral fat area; SMA, skeletal muscle area.

Table S2. Comparison of clinical data and body composition parameters in different disease behavior groups.

|  | Inflammatory group  (n=42) | Complicated group  (n=64) | *p* value |
| --- | --- | --- | --- |
| Demographic Data |  |  |  |
| Male Gender | 27(64.29) | 41(64.06) | 0.981 |
| Age (years) | 26.48±8.58 | 25.98±7.51 | 0.756 |
| BMI (kg/m^2^) | 21.13±3.69 | 19.98±3.20 | 0.090 |
| Disease duration(years), median | 2.00(1.69, 5.00) | 3.00(1.00, 5.00) | 0.677 |
| Location |  |  | 0.194 |
| L1 | 5(11.90) | 11(14.19) |  |
| L2 | 6(14.29) | 3(4.69) |  |
| L3 | 31(73.81) | 50(78.13) |  |
| Perianal | 30(71.43) | 45(70.31) | 0.902 |
| Laboratory Data |  |  |  |
| ESR (mm/h) | 7.50 (5.00, 25.25) | 15.00(6.25, 30.50) | **0.045** |
| CRP (mg/l) | 2.06(0.37, 7.21) | 5.73(1.37, 15.25) | **0.005** |
| Serum Alb (g/l) | 43.92±5.17 | 42.00±4.98 | 0.059 |
| SES-CD | 3.00(2.00, 7.25) | 6.00(2.00, 12.00) | 0.059 |
| Body composition |  |  |  |
| BMI (kg/m^2^) | 21.13±3.69 | 19.98±3.20 | 0.090 |
| SAI (cm^2^/m^2^) | 37.15±16.78 | 30.08±11.94 | **0.015** |
| VAI (cm^2^/m^2^) | 34.53±11.26 | 36.67±11.54 | 0.347 |
| TAI (cm^2^/m^2^) | 71.68±23.32 | 67.19±18.51 | 0.273 |
| SMI (cm^2^/m^2^) | 41.69±7.64 | 37.92±7.17 | **0.011** |
| Visceral obesity |  |  | 0.969 |
| Yes | 8(19.05) | 12(18.75) |  |
| No | 34(80.95) | 52(81.25) |  |
| Decreased skeletal muscle mass |  |  | **0.027** |
| Yes | 26(61.90) | 52(81.25) |  |
| No | 16(38.10) | 12(18.75) |  |

Values are presented as N (%), mean ± SD, or median (interquartile range). BMI, body mass index; ESR, erythrocyte sedimentation rate; CRP, C-reactive protein; Alb, albumin; SAI, subcutaneous adipose index; VAI, visceral adipose index; TAI, total adipose index; SMI, skeletal muscle index.

Table S3. P values for Spearman's correlation analysis of body composition with inflammatory indicators and outcome variables.

| Variables | Decreased skeletal muscle mass | SMI | TAI | Visceral obesity | VAI | SAI | SES-CD | ESR | CRP | Serum Alb | Disease duration | BMI | Sex | Complicated behavior | Age | Moderate to  severe activity | Disease activity |
| --- | --- | --- | --- | --- | --- | --- | --- | --- | --- | --- | --- | --- | --- | --- | --- | --- | --- |
| Decreased skeletal muscle mass | 0.000 | 1.000 | 0.574 | 0.875 | 0.104 | 0.864 | 0.477 | 0.300 | 0.876 | 0.408 | 0.701 | 0.000 | 0.166 | 0.166 | 0.770 | 0.542 | 0.355 |
| SMI | 1.000 | 0.000 | 0.032 | 0.822 | 0.720 | 0.003 | 0.000 | 0.956 | 0.008 | 0.041 | 0.019 | 0.013 | 0.000 | 0.893 | 0.005 | 0.000 | 0.000 |
| TAI | 0.574 | 0.032 | 0.000 | 0.000 | 0.000 | 0.000 | 0.391 | 0.030 | 0.162 | 0.363 | 0.430 | 0.661 | 0.000 | 0.644 | 0.093 | 0.365 | 0.814 |
| Visceral obesity | 0.875 | 0.822 | 0.000 | 0.000 | 0.000 | 0.122 | 0.104 | 0.670 | 0.767 | 0.248 | 0.245 | 0.047 | 0.671 | 0.589 | 0.920 | 0.063 | 0.203 |
| VAI | 0.104 | 0.720 | 0.000 | 0.000 | 0.000 | 0.001 | 0.000 | 0.006 | 0.001 | 0.106 | 0.636 | 0.147 | 0.001 | 0.908 | 0.192 | 0.000 | 0.000 |
| SAI | 0.864 | 0.003 | 0.000 | 0.122 | 0.001 | 0.000 | 0.011 | 0.369 | 0.345 | 0.997 | 0.420 | 0.663 | 0.000 | 0.313 | 0.150 | 0.011 | 0.004 |
| SES-CD | 0.477 | 0.000 | 0.391 | 0.104 | 0.000 | 0.011 | 0.000 | 0.000 | 0.000 | 0.000 | 0.863 | 0.205 | 0.677 | 0.058 | 0.727 | 0.000 | 0.000 |
| ESR | 0.300 | 0.956 | 0.030 | 0.670 | 0.006 | 0.369 | 0.000 | 0.000 | 0.000 | 0.000 | 0.874 | 0.860 | 0.000 | 0.045 | 0.496 | 0.002 | 0.001 |
| CRP | 0.876 | 0.008 | 0.162 | 0.767 | 0.001 | 0.345 | 0.000 | 0.000 | 0.000 | 0.000 | 0.783 | 0.343 | 0.680 | 0.004 | 0.869 | 0.000 | 0.000 |
| Serum Alb | 0.408 | 0.041 | 0.363 | 0.248 | 0.106 | 0.997 | 0.000 | 0.000 | 0.000 | 0.000 | 0.607 | 0.384 | 0.116 | 0.060 | 0.420 | 0.000 | 0.000 |
| Disease duration | 0.701 | 0.019 | 0.430 | 0.245 | 0.636 | 0.420 | 0.863 | 0.874 | 0.783 | 0.607 | 0.000 | 0.038 | 0.605 | 0.679 | 0.003 | 0.739 | 0.687 |
| BMI | 0.000 | 0.013 | 0.661 | 0.047 | 0.147 | 0.663 | 0.205 | 0.860 | 0.343 | 0.384 | 0.038 | 0.000 | 0.498 | 0.182 | 0.036 | 0.363 | 0.385 |
| Sex | 0.166 | 0.000 | 0.000 | 0.671 | 0.001 | 0.000 | 0.677 | 0.000 | 0.680 | 0.116 | 0.605 | 0.498 | 0.000 | 0.982 | 0.133 | 0.258 | 0.728 |
| Complicated behavior | 0.166 | 0.893 | 0.644 | 0.589 | 0.908 | 0.313 | 0.058 | 0.045 | 0.004 | 0.060 | 0.679 | 0.182 | 0.982 | 0.000 | 0.875 | 0.075 | 0.136 |
| Age | 0.770 | 0.005 | 0.093 | 0.920 | 0.192 | 0.150 | 0.727 | 0.496 | 0.869 | 0.420 | 0.003 | 0.036 | 0.133 | 0.875 | 0.000 | 0.997 | 0.471 |
| Moderate to severe activity | 0.542 | 0.000 | 0.365 | 0.063 | 0.000 | 0.011 | 0.000 | 0.002 | 0.000 | 0.000 | 0.739 | 0.363 | 0.258 | 0.075 | 0.997 | 0.000 | 0.000 |
| Disease activity | 0.355 | 0.000 | 0.814 | 0.203 | 0.000 | 0.004 | 0.000 | 0.001 | 0.000 | 0.000 | 0.687 | 0.385 | 0.728 | 0.136 | 0.471 | 0.000 | 0.000 |

SMI, skeletal muscle index; TAI, total adipose index; VAI, visceral adipose index; SAI, subcutaneous adipose index; SES-CD, simple endoscopic score for Crohn’s disease; ESR, erythrocyte sedimentation rate; CRP, C-reactive protein; Alb, albumin; BMI, body mass index.

Table S4. Multivariate analysis of disease activity or moderate-to-severe activity (ESR not included).

|  | disease activity status | | |  | moderate-to-severe disease activity | | |
| --- | --- | --- | --- | --- | --- | --- | --- |
|  | OR | 95%CI | *p* value |  | OR | 95%CI | *p* value |
| SAI | 0.93 | (0.88-0.98) | **0.008** |  | 0.88 | (0.82-0.95) | **0.001** |
| VAI | 1.14 | (1.06-1.22) | **＜0.001** |  | 1.24 | (1.11-1.38) | **＜0.001** |
| SMI | 0.83 | (0.75-0.91) | **＜0.001** |  | 0.86 | (0.78-0.96) | **0.007** |
| Serum Alb (g/l) | 0.82 | (0.65-1.03) | 0.082 |  | 0.82 | (0.66-1.01) | 0.064 |
| CRP (mg/l) | 1.21 | (1.00-1.46) | 0.052 |  | 1.02 | (0.97-1.06) | 0.471 |

SAI, subcutaneous adipose index; VAI, visceral adipose index; SMI, skeletal muscle index; Alb, albumin; CRP, C-reactive protein.

Table S5. Multivariate analysis of disease activity or moderate-to-severe activity (CRP not included).

|  | disease activity status | | |  | moderate-to-severe disease activity | | |
| --- | --- | --- | --- | --- | --- | --- | --- |
|  | OR | 95%CI | *p* value |  | OR | 95%CI | *p* value |
| SAI | 0.92 | (0.87-0.97) | **0.003** |  | 0.87 | (0.81-0.94) | **＜0.001** |
| VAI | 1.15 | (1.07-1.23) | **＜0.001** |  | 1.24 | (1.12-1.38) | **＜0.001** |
| SMI | 0.82 | (0.74-0.90) | **＜0.001** |  | 0.87 | (0.79-0.96) | **0.006** |
| Serum Alb (g/l) | 0.79 | (0.63-0.99) | **0.041** |  | 0.78 | (0.65-0.93) | **0.006** |
| ESR (mm/h) | 1.04 | (0.98-1.11) | 0.211 |  | 1.00 | (0.96-1.05) | 0.887 |

SAI, subcutaneous adipose index; VAI, visceral adipose index; SMI, skeletal muscle index; Alb, albumin; ESR, erythrocyte sedimentation rate.
